# Expression of Concern: Silencing SUMO2 promotes protection against degradation and apoptosis of nucleus pulposus cells through p53 signaling pathway in intervertebral disc degeneration

**DOI:** 10.1042/BSR-20171523_EOC

**Published:** 2020-08-28

**Keywords:** Intervertebral disc degeneration, Nucleus pulposus cells, p53 signaling pathway, Proliferation, SUMO2 gene silencing

The Editorial Office has been made aware of potential issues surrounding the scientific validity of this paper, hence has issued an expression of concern to notify readers whilst the Editorial Office investigates.

